# The Effect of Multisite Phosphorylation on the Conformational Properties of Intrinsically Disordered Proteins

**DOI:** 10.3390/ijms222011058

**Published:** 2021-10-14

**Authors:** Ellen Rieloff, Marie Skepö

**Affiliations:** 1 Division of Theoretical Chemistry, Lund University, P.O. Box 124, SE-221 00 Lund, Sweden; ellen.rieloff@teokem.lu.se; 2 LINXS—Lund Institute of Advanced Neutron and X-ray Science, Scheelevägen 19, SE-223 70 Lund, Sweden

**Keywords:** intrinsically disordered proteins, phosphorylation, force fields

## Abstract

Intrinsically disordered proteins are involved in many biological processes such as signaling, regulation, and recognition. A common strategy to regulate their function is through phosphorylation, as it can induce changes in conformation, dynamics, and interactions with binding partners. Although phosphorylated intrinsically disordered proteins have received increased attention in recent years, a full understanding of the conformational and structural implications of phosphorylation has not yet been achieved. Here, we present all-atom molecular dynamics simulations of five disordered peptides originated from tau, statherin, and β-casein, in both phosphorylated and non-phosphorylated state, to compare changes in global dimensions and structural elements, in an attempt to gain more insight into the controlling factors. The changes are in qualitative agreement with experimental data, and we observe that the net charge is not enough to predict the impact of phosphorylation on the global dimensions. Instead, the distribution of phosphorylated and positively charged residues throughout the sequence has great impact due to the formation of salt bridges. In statherin, a preference for arginine–phosphoserine interaction over arginine–tyrosine accounts for a global expansion, despite a local contraction of the phosphorylated region, which implies that also non-charged residues can influence the effect of phosphorylation.

## 1. Introduction

Intrinsically disordered proteins (IDPs) lack tertiary structure under physiological conditions [1,2], such that they adopt a range of different interchanging conformations rather than a single structure. This is reflected in their rather flat free energy landscapes [3], making them sensitive to environmental changes and post-translational modifications (PTMs), which helps to regulate function. Many IDPs also demonstrate the ability to bind to several targets, and adopt different folds depending on the target. These characteristics of IDPs are advantageous in signaling, regulation, and recognition processes, where IDPs are abundantly involved [4,5].

Phosphorylation is a reversible type of PTM, especially prevalent among intrinsically disordered regions and proteins [6,7,8]. The addition of a bulky phosphoryl group to residues such as serine or threonine adds extra negative charge and enables formation of hydrogen bonds and salt bridges [9], which can induce drastic changes in the conformational ensemble and the dynamics of the IDP. In a simplistic view, assuming electrostatics to be the major determinant of IDP conformation, a net positively charged IDP is expected to contract upon phosphorylation, while a negatively charged or neutral IDP will expand. In a recent atomistic simulation study by Jin and Gräter, this prediction was shown to hold true for multisite phosphorylation of the four peptides studied [10]. Generally, net charge and hydropathy provide good indications of the level of compaction of a protein only in some cases, while many require an additional inspection of the fraction of charged residues and charge pattern, due to their polyampholytic nature [11,12].

In recent years, phosphorylated IDPs have received increased attention [10,13,14,15,16,17,18,19,20,21,22,23]. Changes in global conformation, secondary structure, and local arrangements upon phosphorylation of disordered proteins and regions have been studied experimentally by techniques such as small angle X-ray scattering (SAXS), fluorescence resonance energy transfer, circular dichroism (CD) spectroscopy, and nuclear magnetic resonance (NMR) [13,14,15,20,24,25,26]. Due to the vast conformational ensembles possessed by IDPs, a combination of different techniques is required and often well complemented by atomistic simulations, which through detailed information can provide further insight. After many years of important adjustments, such as refinement of backbone dihedral angles and balancing the water–protein and protein–protein interactions, there are several force field and water model combinations that can be applied to IDPs [27,28]. Less attention has been given to charge–charge interactions, although it has been determined that many standard force fields have a tendency to overestimate salt bridges [29,30]. More recently, it has been shown that, for phosphorylated peptides, this can cause serious discrepancies between simulations and experiments [10,20,31].

In our most recent work involving all-atom molecular dynamics simulations of phosphorylated disordered peptides, Amber ff99SB-ILDN in combination with the TIP4P-D water model showed promising results in describing the conformational ensemble of short disordered peptides [20,31]. Here, we have extended the work with simulations of the non-phosphorylated variants of the four peptides in [31], using the aforementioned force field, and additional analyses of a fifth peptide published in [20], to study the conformational and structural effects upon phosphorylation, with the aim of gaining better insight into the controlling factors. By experimental comparison, we also detect limitations of the force field. Two of the peptides are fragments from the neuroprotein tau, involved in stabilizing neuronal microtubules [32]. Phosphorylation of tau regulates its function, and hyperphosphorylation has been implicated to cause pathological effects by involvement in amyloid fibril formation in Alzheimer’s disease [33,34]. Another two of the peptides are the saliva protein statherin and its fifteen residue long N-terminal fragment, SN15. Statherin maintains a supersaturated environment of calcium phosphate in the saliva, by preventing spontaneous precipitation and crystal growth [35,36,37]. This functionality is closely associated with the N-terminal fragment containing the phosphorylated residues [37]. The last peptide is the 25 residue long N-terminal fragment of β-casein, which naturally contains four phosphorylated serines that sequester calcium and promotes the formation of calcium phosphate nanoclusters [38,39,40].

We observe that, for these peptides, ranging in length from 11 to 43 residues that net charge is not enough to predict the change in global dimensions upon phosphorylation at two to four sites. Instead, salt bridge formation has great impact, depending on the distribution of phosphorylated and positively charged residues throughout the sequence. Furthermore, in statherin, a preference for arginine–phosphoserine interactions over arginine–tyrosine interactions explains the phosphorylation induced changes.

## 2. Results and Discussion

### 2.1. Net Charge Is Not Enough to Explain Phosphorylation Induced Changes

Atomistic simulations of five different disordered peptides in both non-phosphorylated and phosphorylated state, shown in Table 1, have been performed at conditions corresponding to physiological pH (approximately pH 7). The peptides were chosen based on the availability of experimental data and their size, considering computational expense.

**Figure 1:**
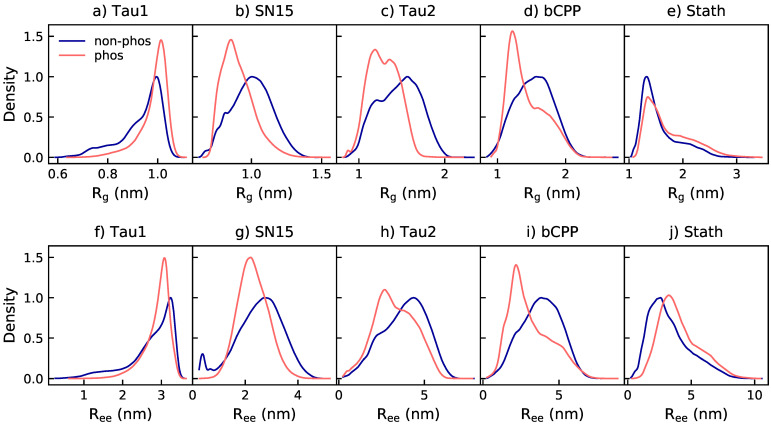
Radius of gyration (R_g_) and end-to-end distance (R_ee_) density distributions of the non-phosphorylated (non-phos) and phosphorylated (phos) variants. The SN15 data are obtained from Ref. [20] (2020 American Chemical Society), and data for the phosphorylated variants of Tau2, bCPP, and Stath from [31].

SN15, Tau2, and bCPP all contract upon phosphorylation, as shown from the peak shift towards lower values of the distributions of radius of gyration (R_g_) and end-to-end distance (R_ee_) in Figure 1, as well as the average values of R_g_ and R_ee_ presented in Table 2. For SN15 and Tau2, the width of the distribution also decreases, while bCPP keeps the same range, only the shape of the distribution changes. Stath and Tau1 both expand, shown from a peak shift towards larger values in the distributions. For Tau1, the expansion is more clear observing the R_g_ distribution than the R_ee_ distribution, which only changes shape by the disappearance of a shoulder at lower values. This, however, causes the average R_ee_, presented in Table 2, to increase. An increase of R_ee_ upon phosphorylation of Tau1 has been detected by fluorescence resonance energy transfer measurements, as reported by Chin et al. [15].

**Figure 2:**
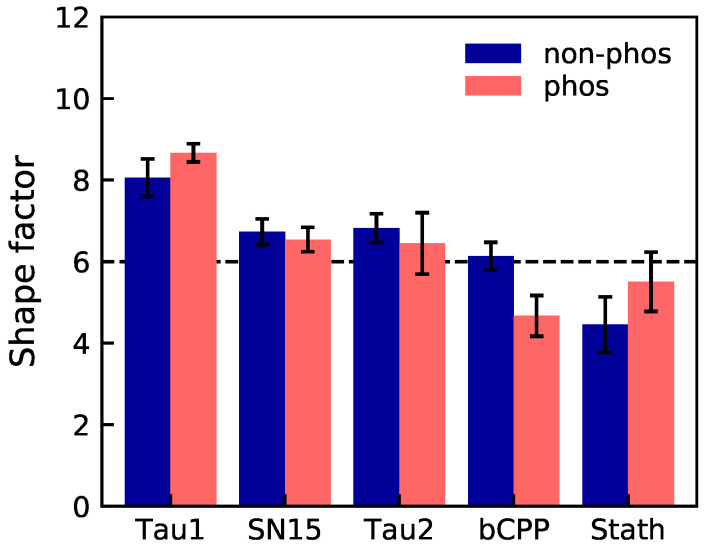
The shape factor of the non-phosphorylated (non-phos) and phosphorylated (phos) variants. The dashed line corresponds to the shape factor of a Gaussian coil. The error bars are based on error propagation of the error estimates determined for radius of gyration and end-to-end distance by block averaging.

The shape factor, presented in Figure 2, can be used as an estimate of the shape of the peptide. If it behaves as a Gaussian coil, the shape factor is approximately 6, whereas for a stiff rod, it is around 12. SN15, Tau2, and bCPP are shown to behave rather coil-like in non-phosphorylated state, while Tau1 is more stiff, and Stath more contracted. Upon phosphorylation, bCPP becomes more contracted than a Gaussian coil, while Stath expands to become more coil-like.

Comparing the induced changes of R_g_ and R_ee_ with the net charge of the non-phosphorylated peptides, it is clear that the prediction of Jin and Gräter, i.e., that net charge controls the effect of phosphorylation [10], only holds for SN15, Tau2, and Stath. bCPP contracts despite having a negative net charge, and Tau1 expands despite the positive net charge. Note that the peptides in this study are distinctly shorter (11–43 residues) compared to the IDPs in the study by Jin and Gräter (approximately 80 residues) [10], hence local interactions are expected to have a more direct effect on the global dimensions. To understand the effect of phosphorylation of these peptides, we therefore need to investigate changes in secondary structure and specific interactions.

### 2.2. Phosphorylation of Tau1 Favors Expanded Conformations

**Figure 3:**
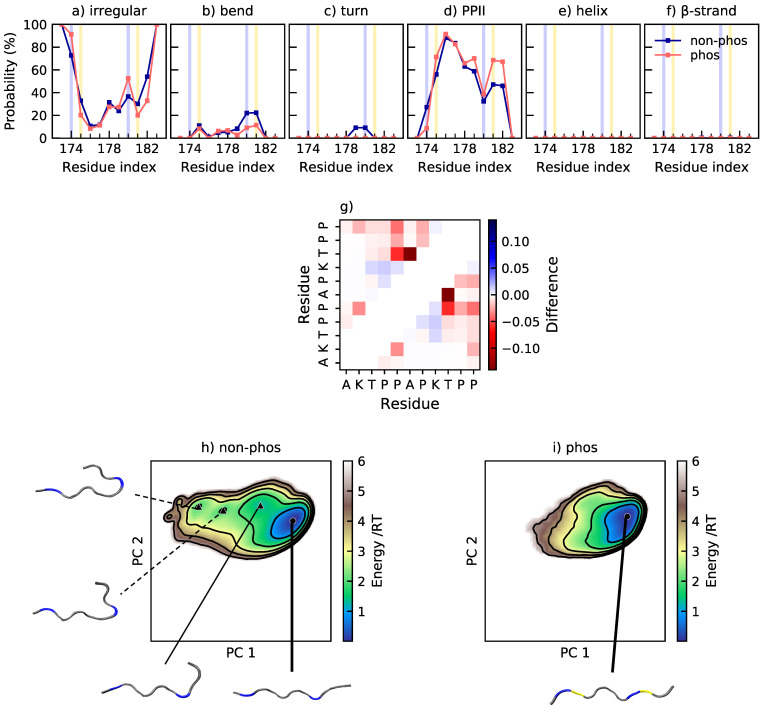
(**a**–**f**) Secondary structure content along the non-phosphorylated and phosphorylated sequence of Tau1. The helix includes α-helix and 3_10_-helix. β-strand also includes β-bridge. The positions of phosphorylated and positively charged residues are highlighted in yellow and blue, respectively; (**g**) change in contact probability upon phosphorylation of Tau1; (**h**,**i**) energy landscapes and conformations in minima of non-phosphorylated and phosphorylated Tau1. The energy landscapes are constructed using the first two components from principal component analysis, applying the same basis set for both variants. Hence, they are directly comparable. Contour lines are drawn for integer energy levels in the interval 1≤RT≤5 and the minimum of each basin is represented by a marker depending on the energy: ●: ≤1RT, ▲: ≤2RT. A thick line corresponds to the most populated basin, while dashed lines to the least populated basins. In the conformations, positively charged residues are shown in blue, and phosphorylated residues in yellow.

Tau1 is dominated by irregular structure and polyproline type II helix (PPII), as shown in Figure 3a–f. It possesses 46% and 51% PPII in the non-phosphorylated and phosphorylated state, respectively. Elam et al. [41] have predicted close to 50% PPII content in this region of Tau, and CD measurements of this segment indicate an increase of PPII content upon phosphorylation [15]. In Figure 3a–f, it is shown that all structural changes upon phosphorylation at T175 and T181 take place at the C-terminal end of the peptide, from residue 179 and forward. The propensity for bends and turns at residue 179–181 decreases, while the PPII content increases at residues 181–182. There is occasional salt bridge formation between the phosphothreonines and their respective neighboring lysine. Specifically, the probability of salt bridge formation is 7±2% for pT175–K174 and 9±2% for pT181–K180. The most occurring salt bridge is, however, formed between pT175 and the N-terminal, with a probability of 49±9%. However, due to the close proximity between the salt bridging residues, the effect on the overall dimensions of the peptide is small. Since Tau1 is a short and rather stiff peptide, as shown by the shape factor in Figure 2, there is limited contact between residues. The change in contact probability upon phosphorylation is also small, according to Figure 3g, which reveals that the main change is a decrease of contact between T181 and the preceding residues A177 and P178, in agreement with the decreased probability of a bend or turn in that region, as shown by Figure 3b,c. The conformational effects of phosphorylation of Tau are well summarized by Figure 3h,i, showing the energy landscape and conformations of non-phosphorylated and phosphorylated Tau1. The energy landscape of non-phosphorylated Tau1 contains several minima, of which the minimum containing expanded conformations dominate, in line with the relatively high shape factor. Other less populated minima contain conformations with a kink in the C-terminal end, originating from a bend or turn. Upon phosphorylation, the minima with kinked conformations disappears, leaving only the minima with expanded conformations. This is in line with decreased contact probability and explains the change in shape of the R_g_ and R_ee_ distributions, from a peak with a preceding shoulder to a single peak.

### 2.3. Phosphorylation Increases the Helix Propensity and Induces Salt Bridge Formation in Tau2 and SN15

**Figure 4:**
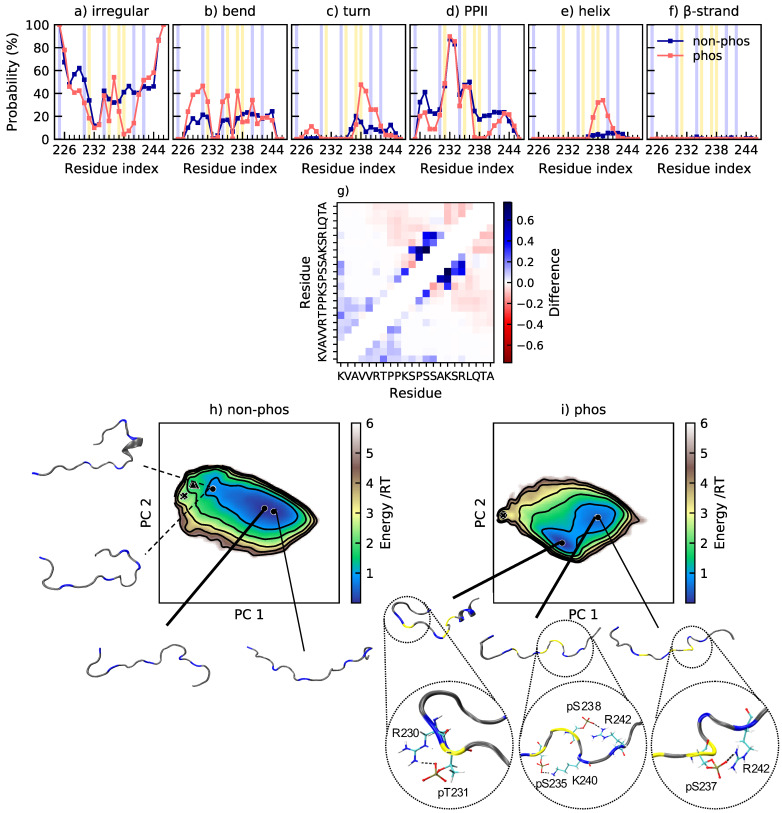
(**a**–**f**) Secondary structure content along the non-phosphorylated and phosphorylated sequence of Tau2. Helix includes α-helix and 3_10_-helix. β-strand also includes β-bridge. The data for the phosphorylated peptide are previously published in [31]. The positions of phosphorylated and positively charged residues are highlighted in yellow and blue, respectively; (**g**) change in contact probability upon phosphorylation of Tau2; (**h**,**i**) energy landscapes and conformations in minima of non-phosphorylated and phosphorylated Tau2. The energy landscapes are constructed using the first two components from principal component analysis, using the same basis set for both variants, hence making them directly comparable. Contour lines are drawn for integer energy levels in the interval 1≤RT≤5 and the minimum of each basin is represented by a marker depending on the energy: ●: ≤1RT, ▲: ≤2RT, ✖: ≤3RT. Thick lines correspond to the most populated basins, while dashed lines to the least populated basins. In the conformations, positively charged residues are shown in blue and phosphorylated residues in yellow.

**Figure 5:**
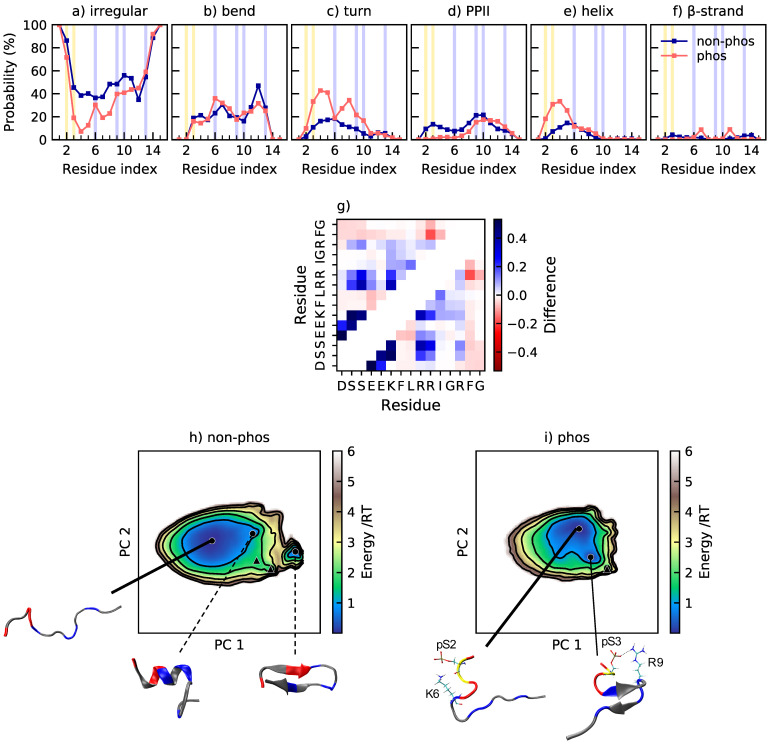
(**a**–**f**) Secondary structure content along the non-phosphorylated and phosphorylated sequence of SN15. Helix includes α-helix and 3_10_-helix. β-strand includes also β-bridge. These data are obtained from Ref. [20] (2020 American Chemical Society). The positions of phosphorylated and positively charged residues are highlighted in yellow and blue, respectively; (**g**) change in contact probability upon phosphorylation of SN15, based on data from Ref. [20]. (**h**,**i**) Energy landscapes and conformations in minima of non-phosphorylated and phosphorylated SN15. The energy landscapes are constructed using the first two components from principal component analysis, using the same basis set for both variants. Hence, they are directly comparable. Contour lines are drawn for integer energy levels in the interval 1≤RT≤5 and the minimum of each basin is represented by a marker depending on the energy: ●: ≤1RT, ▲: ≤2RT. A thick line corresponds to the most populated basin, while dashed lines to the least populated basins. In the conformations, positively charged residues are shown in blue, negatively charged residues in red, and phosphorylated residues in yellow. Phosphorylated and positively charged residues that are close are shown explicitly.

Tau2 and SN15 are both mainly irregular and report an increase of helicity upon phosphorylation, see Figure 4a–f and Figure 5a–f, respectively. The helical region is identified as “pSpSAKSR” in Tau2 and “pSpSEEKFLR” in SN15, according to Figure 4e and Figure 5e. The sequences, hence, share two characteristics: (1) the helical region starts with two phoshorylation sites, and (2) three or four steps away from the phosphorylation site, a positively charged residue is positioned. Phosphorylation has been shown to stabilize α-helices if the phosphorylation site is located in the N-terminal end of the helix, by electrostatic interaction between phosphorylated serines and the macrodipole of the helix, and by hydrogen bonding with the amide backbone [42]. With a i,i+4 spacing between a phosphorylated serine and a lysine, phosphorylation also stabilizes α-helices through salt bridge formation between the side groups [43].

For Tau2, a phosphorylation-induced increase of α-helical structure from 5 to 40% in region A239–R242 has been reported [13]. In these simulations, the main helical increase upon phosphorylation is associated with region S237–K240, where the increase is from 4 to 26%. However, the helical increase is mainly due to 3_10_-helix, since the increase of α-helix is only from 1 to 5%. Hence, the simulations are in qualitative agreement with the experiments, but the quantitative results should be treated with caution. In addition, in SN15, the larger part of the helical increase is due to 3_10_-helix, and an increase of α-helix is supported by CD spectroscopy [20], once again giving qualitative support to the findings in this study. Notice also that, while it is hard to make quantitative comparisons with CD data, our study on SN15 suggested that the simulations underestimate the structural content [20], which is the same as observed for Tau2.

While helix formation decreases the R_g_ and R_ee_, salt bridge formation can also contribute to the compaction observed upon phosphorylation. In Tau2, several salt bridges have been established from NMR measurements, specifically pT231–R230, pS237–K240, and pS238–R242 [13]. pT231–R230 and pS238–R242 are indeed the two most occurring salt bridges according to Table 3, while pS237–R242 is the third most common. Apart from the increase of helical content related to phosphorylation, Figure 4b reveals an interesting pattern of bends after phosphorylation, where the charged residues R, K, pT, and pS are enriched in bends. The conformations in Figure 4 illustrate how the salt bridges contribute to the formation of bends. Since the probability of a turn at A227–V229 is roughly the same as the probability of the pT231–K225 salt bridge (see Figure 3 and Figure 4c), and V228 is located right between K225 and pT231, we conclude that this turn is also a result of a salt bridge interaction. Hence, this peptide shows that salt bridge formation can induce bends and turns.

Comparing the energy landscapes of non-phosphorylated and phosphorylated Tau2 in Figure 4h,i, it is shown that, for both peptides, more extended conformations, such as in the minima furthest to the right, are sampled, but to a different extent. These type of conformations are more common in the non-phosphorylated variant, while the most populated basin contains conformations with the N-terminal end folded over, to come closer to the phosphorylated residues. While K225 rarely involves in a proper salt bridge with other residues than pT231, it is still energetically favorable to be in rather close vicinity of the phosphorylated region, considering both the charged side chain and the N-terminus. These types of conformations give rise to an increased contact probability within the N-terminal part of the chain, see Figure 4g. The increased contact probability close to the diagonal in the middle to C-terminal end corresponds to the increase of helical structure and certain salt bridges. Apart from those, there is a decrease of the probability of contacts within the C-terminal end upon phosphorylation. The two minima in the left part of the energy landscape of non-phosphorylated Tau2 in Figure 4h are examples of conformations with a higher level of contact within the C-terminal end. They originate from the electrostatic attraction between the C-terminus and the positively charged residues. In phosphorylated Tau2, that region of the energy landscape is visited much less (see Figure 4i), in agreement with the changes in contact probability. Notice, however, that the probability of conformations with one end folded over is much higher after phosphorylation, which explains the decrease in R_g_ and R_ee_. The conformation corresponding to the minimum in the most populated basin for the phosphorylated peptide additionally shows a helix in the C-terminal end, which also contributes to a decreased R_g_ and R_ee_.

In SN15, the salt bridges pS2–K6, pS3–K6, pS3–R9, and pS3–R10 are the most probable and all form with an approximately 25% occurrence. From the change in contact probability displayed in Figure 5g, it appears that the pS2–K6 and pS3–K6 salt bridges contribute to stabilize the formed helix. The pS3–R9 and pS3–R10 salt bridges are also visible in the contact map and contribute to an increase in the amount of more compact conformations after phosphorylation. In the energy landscape in Figure 5, it is shown that phosphorylation shifts the position of the main minima in the energy landscape, from an area of more coil-like structures to a more compact state. The non-phosphorylated peptide also samples conformations that are more compact with a higher content of secondary structure, but more rarely than the phosphorylated peptide. The conformation corresponding to the minimum in the most populated basin in the phosphorylated peptide has residue pS2 and K6 close enough to be in contact; however, there is no helix, but instead a turn at residues E4–E5. This shows that it is favorable to have pS2 and K6 in contact, but that the interaction does not necessarily imply helix formation. In Figure 5c, it was shown that the turn content in region S3–E5 also increases upon phosphorylation, not only the helix content. There is also an increase of turn content in region F7–R11, which is partly caused by occasional β-strand formation, as shown in the other conformation in Figure 5, and partly by residues pS3 and R9 coming close to form a salt bridge, in line with the turn induced in Tau2. Both of these changes give rise to more compact conformations. We must, however, note that SAXS measurements have indicated that a compaction upon phosphorylation is plausible, but probably smaller than shown in the simulations [20]. While Jin and Gräter found that changes in the hydration shell upon phosphorylation can hide global conformational changes in SAXS measurements, they also concluded that the force field used in this study overestimates the charge effect, thus providing two different explanations of the deviations between the simulations and experiments [10]. Note also that the contact map reports a decrease of contact between R10 and F14, a contact probably formed due to cation–π interaction, which will be discussed further in the section regarding Stath.

### 2.4. Salt Bridge Formation Shifts the Conformational Ensemble of bCPP

**Figure 6:**
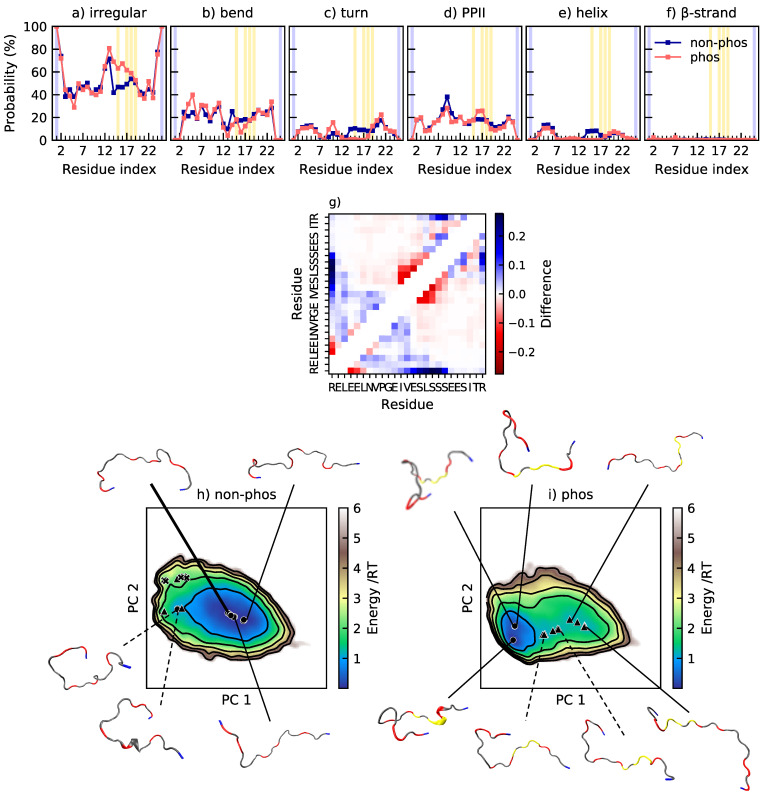
(**a**–**f**) Secondary structure content along the non-phosphorylated and phosphorylated sequence of bCPP. Helix includes α-helix and 3_10_-helix. β-strand includes also β-bridge. The data for the phosphorylated peptide are previously published in [31]. The positions of phosphorylated and positively charged residues are highlighted in yellow and blue, respectively; (**g**) change in contact probability upon phosphorylation of bCPP; (**h**,**i**) energy landscapes and conformations in minima of non-phosphorylated and phosphorylated bCPP. The energy landscapes are constructed using the first two components from principal component analysis, using the same basis set for both variants. Hence, they are directly comparable. Contour lines are drawn for integer energy levels in the interval 1≤RT≤5 and the minimum of each basin is represented by a marker depending on the energy: ●: ≤1RT, ▲: ≤2RT, ✖: ≤3RT. A thick line corresponds to the most populated basin, while dashed lines to the least populated basins. In the conformations, positively charged residues are shown in blue, negatively charged residues in red and phosphorylated residues in yellow.

For bCPP, the secondary structure content is dominated by an irregular structure and is highly similar in phosphorylated and non-phosphorylated states, as shown by Figure 6a–f, in agreement with CD spectroscopy results by Farrell et al. [25]. The small difference that occurs upon phosphorylation at S14, S17, S18, and S19 is a change from helix and turn to irregular structure in region E14–S17. The vanishing of helical content is in agreement with the conclusion of Andrew et al. that phosphorylation of a residue in the interior of a helix, without a positively charged residue within suitable distance, destabilizes the helix [42]. Since disruption of a short helix would not cause a contraction of the peptide, the conformational changes in bCPP upon phosphorylation are not explained by secondary structure. Instead, the contraction is due to electrostatic attraction including salt bridge formation between the positively charged end residues and the phosphorylated residues, as seen in Table 4. Although both end residues are arginines, there is a preference of R1 to interact with the phosphorylated region over R25, due to the respective charges of the termini. This is evident from the fact that the N-terminus is also involved in salt bridges with the phosphorylated residues, and further shown by the difference in contact probability in Figure 6g. When R1 interacts with the phosphorylated residues, it causes the peptide to fold over, reducing R_g_ and R_ee_ substantially. From the energy landscapes in Figure 6h,i, it is shown that before phosphorylation the minima with lowest energy contain more extended conformations, while after phosphorylation the minima with lowest energy instead showcase the N-terminal part being folded over.

Based only on the net charge of non-phosphorylated bCPP, it was expected that it would expand upon phosphorylation. Considering only region E13–E21, which contains the four phosphorylation sites, this effect was noticed. The average distance between the C atoms of residue 13 and 21 increases from 1.91±0.03 nm to 2.12±0.03 nm upon phosphorylation. However, due to the strong electrostatic interaction between the arginines and the phosphorylated region that are far apart in the sequence, the global result is compaction. Hence, the relative position of charged residues is very important to consider for the effects of phosphorylation on the overall dimensions of the peptide.

We previously showed that the addition of 150 mM NaCl had negligible effects on the salt bridges and global conformational properties of phosphorylated bCPP [31]. The same applies to non-phosphorylated bCPP, as presented in Supplementary File S1, Figures S1 and S2. However, although the average values of R_g_ at 0 and 150 mM are within error, there is a slight increase in the phosphorylated variant and decrease in the non-phosphorylated variant, see Table 5. Hence, at 150 mM NaCl, the difference observed in R_g_ between the two variants vanishes, considering the associated error. Note, however, that the distributions still have distinctly different shapes, hence we argue that the conformational ensembles are still different. The same trend is observed in the average R_ee_ values, although a difference with respect to phosphorylation state still remains at 150 mM NaCl, see Table 5. In addition, in the calculated scattering curve (Supplementary File S1, Figure S2), the effect of salt is smaller than the effect of phosphorylation. The difference between the form factor of non-phosphorylated and phosphorylated bCPP is, however, still rather small, so we suspect that it can be hard to detect experimentally with SAXS. Based on the fraction of charged residues and level of charge separation, we expect the other peptides in this study to show smaller effects in regard to salt concentration than bCPP. Hence, we expect the results observed here to be also valid at 150 mM NaCl.

### 2.5. Arginine—Phosphoserine Interactions Outshines Arginine—Tyrosine Interactions in Stath

**Figure 7:**
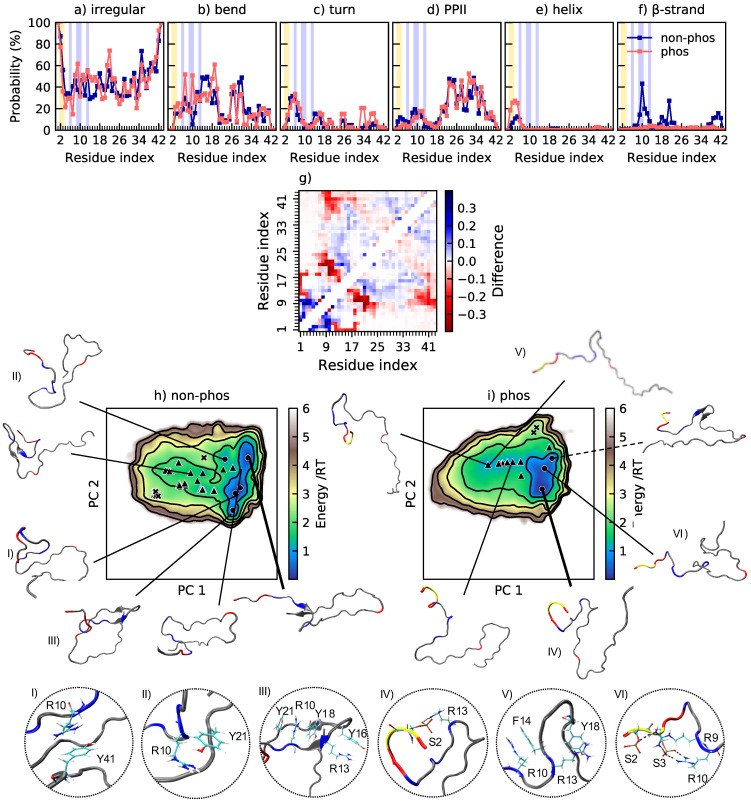
(**a**–**f**) Secondary structure content along the non-phosphorylated and phosphorylated sequence of Stath. Helix includes α-helix and 3_10_-helix. β-strand includes also β-bridge. The data for the phosphorylated peptide are previously published in [31]. The positions of phosphorylated and positively charged residues are highlighted in yellow and blue, respectively; (**g**) change in contact probability upon phosphorylation of Stath; (**h**,**i**) energy landscapes and conformations in minima of non-phosphorylated and phosphorylated Stath. The energy landscapes are constructed using the first two components from principal component analysis, using the same basis set for both variants, hence making them directly comparable. Contour lines are drawn for integer energy levels in the interval 1≤RT≤5 and the minimum of each basin is represented by a marker depending on the energy: ●: ≤1RT, ▲: ≤2RT, ✖: ≤3RT. A thick line corresponds to the most populated basin, while a dashed line to the least populated basin. In the conformations, positively charged residues are shown in blue, negatively charged residues in red, and phosphorylated residues in yellow. The circles show specific interactions within the peptide in the conformations corresponding to the Roman numerals.

Upon phosphorylation of Stath, the three largest changes in secondary structure are a decrease of β-strand structure, an increase of helical structure, and an increase of turns, according to Figure 7a–f. The increase of helical structure is in the same region as observed for the N-terminal fragment SN15. Figure 7f implies that residues R10, Y18, Y21, and Y41 are of extra importance for the formation of β-sheet. The cation–π interaction that can occur between aromatic residues, such as tyrosine, and cationic residues, such as arginine, have been shown to be common in proteins [44]. A correlation between β-strands and cation–π interactions have also been established [45]. Table 6 show that the cation–π interaction indeed is more occurring in non-phosphorylated Stath than in phosphorylated Stath, suggesting that it drives the formation of β-strands. The conformations in Figure 7I–III show examples of the cation–π interaction in non-phosphorylated Stath. Although the aromatic–cation interactions are more common in non-phosphorylated Stath, they still occur in phosphorylated Stath, as exemplified by Figure 7. Upon phosphorylation, the occurrence of cation–π interaction decreases substantially, while salt bridge formation appears according to Table 7. Notice that R10, which was shown to interact with tyrosines, is involved in one of the most probable salt bridges, pS3–R10. Hence, the arginine–phosphoserine interaction is deemed stronger than the arginine–tyrosine interaction. The replacement of arginine–tyrosine interaction with arginine–phosphoserine causes the β-strands to vanish, which explains the observed expansion.

As presented above, SN15, which is the first fifteen residues of Stath, contracts upon phosphorylation, which was explained by the increased helicity and formation of salt bridges. Supplementary File S1, Figure S3 shows that, in phosphorylated Stath, the global dimensions of the first fifteen residues, Stath_1–15_ agree with those of the fragment (SN15). In the non-phosphorylated variant, the distributions are also rather similar, except for a sharp peak in both the R_g_ and R_ee_ distributions, which corresponds to a basin in the energy landscape with the conformation shown in Supplementary File S1, Figure S3c. Regarding the secondary structure, according to Supplementary File S1, Figure S4, the largest difference between SN15 and Stath_1–15_ is caused by β-strand not forming in SN15, due to lacking its partner further on in the sequence. There are also some differences in bends and turns, but the increase of helical propensity is similar. Hence, overall, the first fifteen residues of Stath behave rather similarly in the full peptide and as a standalone fragment, although especially the presence of the rest of the sequence induces β-strand formation. Despite this discrepancy, we can conclude that phosphorylation of Stath causes a contraction of the first fifteen residues, but an expansion of the full peptide, due to disruption of β-sheets.

## 3. Conclusions

Some of the peptides in this study contracted upon phosphorylation, while others became more expanded. However, the net charge was not enough to predict the effect in these short peptides. Instead, we have identified factors that appeared to be of greater importance, of which the first is the distribution of charged residues, in line with the influence of linear charge distribution on the conformational ensemble of IDPs [46]. Especially the relative position of phosphorylated and positively charged residues mattered, considering that salt bridges formed between residues far from each other in the sequence had the largest effect on the overall dimensions of the peptide. Regarding salt bridges, Kumar et al. have shown that phosphorylation can re-wire salt bridges by competing with already present E–R salt bridges [47], but no such tendencies were observed for these peptides. Here, the possible salt bridges in the non-phosphorylated peptides were either low in probability or did not change much upon phosphorylation. In Stath, competitive interactions between positively charged residues, aromatic residues, and phosphorylated residues accounted for the changes upon phosphorylation. This shows that, for peptides which include arginine, it can be of importance to also consider aromatic residues. In both bCPP and Stath, phosphorylation induced the opposite effect on the local and global dimensions, hence, to understand the purpose/implications of the phosphorylated residues, both length-scales should be studied. This is especially important dealing with longer IDPs where local/non-local effects can have larger compensatory effect than observed for short peptides [14].

Regarding secondary structure, the separation between phosphorylated and positively charged residues was shown to control the helix propensity, and salt bridges additionally induced changes in the amount of bends and turns. Comparison with experimental data on secondary structure for SN15 and Tau2 indicates that the simulations underestimate the structural content. For these peptides, a preference of 3_10_- over α-helix was also observed, while the experimental data only considered α-helix. Hence, the simulations were better at indicating trends than producing exact measurements of secondary structure. Overall, the simulation results were often in qualitative agreement with available experimental data, suggesting that, despite the deficiency related to secondary structure and the reported tendency of the force field to overestimate charge–charge interactions, simulations with this force field can still contribute to an increased understanding of the implications of phosphorylation.

As a final note, this study shows that there are several factors contributing to the outcome of phosphorylation, and that they are of varying importance in different peptides. This shows that phosphorylation indeed is complex; however, it is still possible to obtain a better understanding of these factors individually. Therefore, we have an ongoing project in which the number of phosphorylated residues and their positions are varied in a controlled manner, to investigate the effects of those factors systematically.

## 4. Materials and Methods

All-atom molecular dynamics simulations of the systems shown in Table 8 were performed using GROMACS version 2018.4 (version 4.6.7 for simulation of Stathn) [48,49,50,51,52] with the AMBER ff99SB-ILDN [53] force field and the TIP4P-D [54] water model. Parameters for phosphorylated residues were derived from Homeyer et al. [55] and Steinbrecher et al. [56]. Please note that some of the data sets are previously published and only re-analyzed for this study.

Initial configurations of the peptides were constructed from the sequence as linear chains using Avogadro 1.2.0 [57], optimizing the structure with the auto-optimization tool. SN15n and Stathn were constructed as linear chains in PyMOL [58]. Each peptide was placed in a rhombic dodecahedron box with a minimum distance between the peptide and the box edges of 1 nm, and solvated. The number of water molecules is specified in Table 8, alongside the number of chloride and sodium ions that were added to neutralize the system and in two cases obtain a salt concentration of 150 mM. Periodic boundary conditions were employed in all directions. The equations of motion were integrated using the Verlet leapfrog algorithm [59] with a time step of 2 fs. Non-bonded interactions were treated with a Verlet list cutoff scheme. The short-ranged interactions were calculated using neighbor lists with a cutoff of 1 nm. Long-ranged dispersion corrections were applied to energy and pressure and long-ranged electrostatic interactions were treated by Particle Mesh Ewald [60] with a cubic interpolation and 0.16 nm grid spacing. All bond lengths were constrained using the LINCS algorithm [61]. Solute and solvent were separately coupled to temperature baths at 298 K using the velocity rescaling thermostat [62] with a 0.1 ps relaxation time. Parrinello–Raman pressure coupling [63] was used to keep the pressure at 1 bar, using a 2 ps relaxation time and $4.5\cdot 10^{-5}$ bar^-1^ isothermal compressibility.

Energy minimization was performed by the steepest descent algorithm until the system was converged within the available machine precision. Initiation of five replicates per system with different starting seeds were performed separately in two steps using position restraints on the peptide. The first step was 500 ps of NVT simulation (constant number of particles, volume, and temperature) performed to stabilize the temperature, followed by the second step of 1000 ps of NPT simulation (constant number of particles, pressure, and temperature) to stabilize the pressure. Production runs of the five replicates per system were performed in the NPT ensemble, for at least 1 µs per replicate. bCPPp with 150 mM salt was simulated in 10 replicates for 0.7 µs each. The total simulation time per system is stated in Table 8. Energies and coordinates were saved every 10 ps, except for in the simulations with 150 mM NaCl. The saving frequency there was every 50 or 40 ps, for bCPPn and bCPPp, respectively.

### Analysis

R_g_ and R_ee_ were calculated using GROMACS 2018.4 and the *gmx analyze* routine was used to obtain averages and error estimates from block averaging analysis. Distributions were obtained by Gaussian kernel estimation using the SciPy package version 1.5.4 [64]. The shape factor, $r_{s}$, was calculated from the average values of R_g_ and R_ee_ according to:

(1) $$r_{s}=\frac{R_{\mathrm{ee}}^{2}}{R_{g}^{2}}.$$

Secondary structure was determined using the DSSP program version 2.2.1 [65] with an extension to detect polyproline type II structure [66,67], on 10,000 equally spaced frames from the combined trajectory. The MDTraj Python library version 1.9.3 [68] was used to obtain contact maps, analyze salt bridges, and cation–π interactions. For the contact maps, contact was defined as when two atoms of different residues were within 0.4 nm of each other. Since salt bridges are formed as a result of hydrogen bonding and electrostatic interactions, they have been assessed by analyzing the presence of hydrogen bonds based on the criterion in reference [69], as implemented in MDTraj. Cation– interactions were analyzed based on the position of the NZ atom in arginine and CG and CZ in tyrosine. Interaction was defined to occur when both the distances R:NZ–Y:CG and R:NZ–Y:CZ were ≤0.6 nm [44]. The energy landscapes were calculated using principal component analysis following the approach described by Campos and Baptista [70], with the differences described by Henriques et al. [71]. In short, principal component analysis was applied to the Cartesian coordinates of the backbone atoms of the protein, obtained after translational and rotational least square fitting on the central structure of the simulation. The conditional free energy was calculated from the probability density function in the representation space constructed by the first two principal components, obtained by Gaussian kernel density estimation. Snapshots from the simulations were produced using VMD 1.9.3 [72,73,74]. Data were plotted using a Jupyter Notebook [75] with Python version 3.6.4 and packages NumPy version 1.19.5 [76] and Matplotlib version 2.1.2 [77].

Convergence and sampling quality were assessed by comparing the R_g_ and R_ee_ distributions, and energy landscapes, between the replicates, as well as by observing the auto-correlation function and convergence of the block average error estimate of R_g_ and R_ee_ in the concatenated simulation. These data are available in Supplementary File S2.

## Figures and Tables

**Table 1:** Full name and sequence of the peptides included in this study. Positively charged residues are marked in blue, negatively charged in red, and phosphorylation sites are highlighted with yellow. The number of residues (N_res_), net charge of the non-phosphorylated variant (Z_no-phos_), and the phosphorylated variant (Z_phos_) are also shown.

Name	Peptide	Sequence	N_res_	Z_no-phos_	Z_phos_
Tau1	Tau_173–183_	AKTPPAPKTPP	11	+2	−2
SN15	Statherin_1–15_	DSSEEKFLRRIGRFG	15	+1	−3
Tau2	Tau_225–246_	KVAVVRTPPKSPSSAKSRLQTA	22	+5	−3
bCPP	-casein_1–25_	RELEELNVPGEIVESLSSSEESITR	25	−5	−13
Stath	Statherin	DSSEEKFLRRIGRFGYGYGPYQPVPEQPLYPQPYQPQYQQYTF	43	0	−4

**Table 2:** Average radius of gyration (R_g_) and end-to-end distance (R_ee_) of the non-phosphorylated (non-phos) and phosphorylated (phos) variants. Data for SN15 are obtained from [20] and for the phosphorylated peptides of Tau2, bCPP, and Stath from [31].

	Radius of Gyration (nm)		End-to-End Distance (nm)	
**Peptide**	**non-phos**	**phos**	**non-phos**	**phos**
Tau1	0.93±0.01	0.98±0.01	2.74±0.06	2.89±0.02
SN15	1.00±0.01	0.90±0.01	2.54±0.09	2.30±0.03
Tau2	1.46±0.02	1.29±0.03	3.83±0.09	3.27±0.17
bCPP	1.53±0.03	1.43±0.03	3.80±0.08	3.09±0.15
Stath	1.56±0.04	1.73±0.09	3.30±0.24	4.05±0.17

**Table 3:** Probability of salt bridge formation (%) between phosphorylated residues and positively charged residues in Tau2, where NT is the N-terminus. The data are obtained from Ref. [31]. The values printed in bold correspond to the experimentally established salt bridges [13].

Residue	NT	K225	R230	K234	K240	R242
pT231	1±1	10±3	37±10	3±2	∼0	∼0
pS235	<1	2±1	<1	15±4	17±2	6±3
pS237	2±1	4±3	3±10	17±2	19±2	29±2
pS238	4±1	5±2	3<1	∼0	5±4	35±6

**Table 4:** Probability of salt bridge formation (%) between phosphorylated residues and positively charged residues in bCPP, where NT is the N-terminus. The data are obtained from Ref. [31].

Residue	NT	R1	R25
pS15	2±1	6±1	2±1
pS17	3±1	7±1	7±2
pS18	4±1	13±4	12±4
pS19	1±1	10±4	15±4

**Table 5:** Average radius of gyration and end-to-end distance of the non-phosphorylated (non-phos) and phosphorylated (phos) bCPP in the presence of 0 and 150 mM NaCl. The data for the phosphorylated peptide are previously published in [31].

	Radius of Gyration (nm)		End-to-End Distance (nm)	
	0 mM	150 mM	0 mM	150 mM
non-phos	1.53±0.03	1.48±0.02	3.80±0.08	3.64±0.09
phos	1.43±0.03	1.45±0.03	3.09±0.15	3.37±0.13

**Table 6:** Probability of cation–π interaction (%) for certain pairs of residues in non-phosphorylated (non-phos) and phosphorylated (phos) Stath.

Residues	Non-phos	phos
R10–Y18	13.8±6.3	1.6±0.9
R10–Y21	32.0±8.6	3.9±0.7
R10–Y41	9.2±4.3	0.4±0.2

**Table 7:** Probability of salt bridge formation (%) between phosphorylated residues and positively charged residues in Stath, where NT is the N-terminus. The data are obtained from Ref. [31].

Residue	NT	K6	R9	R10	R13
pS2	<1	23±7	23±8	12±1	8±1
pS3	12±3	9±1	30±8	32±7	6±3

**Table 8:** Details of the simulations included in this work. The suffix n stands for non-phosphorylated peptide, while the suffix p stands for phosphorylated.

Peptide	Box Volume (nm^3^)	Number of	Number of	Number of	Total Simulation
		Solvent Molecules	Sodium Ions	Chloride Ions	Length (µs)
Tau1n	157.63	5130	0	2	10.0
Tau1p	140.55	4594	2	0	5.0
Tau2n	724.974	23862	0	5	6.0
SN15n ^a^	272.13	8839	0	1	14.4
SN15p ^a^	294.52	9703	3	0	22.0
Tau2p ^b^	722.941	23816	3	0	11.0
bCPPn	1009.24	32975	5	0	5.0
bCPPn, 150 mM	1009.24	32793	96	91	5.0
bCPPp ^b^	1002.41	32815	13	0	6.0
bCPPp, 150 mM ^b^	1002.41	32633	104	91	7.0
Stathn ^c^	930.47	30651	0	0	17.0
Stathp ^b^	942.11	30942	4	0	12.0

^a^ Previously published [20]. ^b^ Previously published [31]. ^c^ Using GROMACS version 4.6.7.

## Data Availability

Data are included in the article or Supplementary Material.

## Supplementary Materials

The following are available online at https://www.mdpi.com/article/10.3390/ijms222011058/s1.

## Abbreviations

The following abbreviations are used in this manuscript:

IDP	Intrinsically disordered protein
CD	Circular dichroism
NMR	Nuclear magnetic resonance
R_g_	Radius of gyration
R_ee_	End-to-end distance
SAXS	Small-angle X-ray scattering
