# Supplementary material for: The Effect of Multisite Phosphorylation on the Conformational Properties of Intrinsically Disordered Proteins
Source: Int J Mol Sci. 2021 Oct 14;22(20):11058. doi: 10.3390/ijms222011058 (PMC8541499; doi:10.3390/ijms222011058)
Supplement: Supplementary file 1 [file ijms-22-11058-s001.zip › SupplementaryMaterial/SupplementaryFileS1.pdf]

Supplementary File S1 for Conformational effects of multisite phosphorylation of disordered peptides

E. Rieloff[1] and M. Skepö[1,2*]

[1] Division of Theoretical Chemistry, Lund University, Lund, Sweden
[2] LINXS - Lund Institute of Advanced Neutron and X-ray Science, Lund, Sweden

* marie.skepo@teokem.lu.se

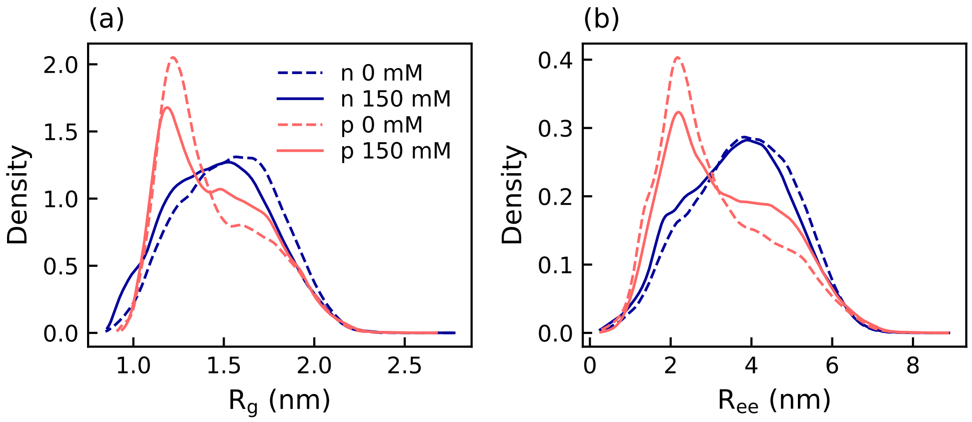

Figure S1: Distribution of a) radius of gyration and b) end-to-end distance of non-phosphorylated (n) and phosphorylated (p) bCPP simulated with 0 or 150 mM NaCl. The data for the phosphorylated peptide are taken from ref. [1].

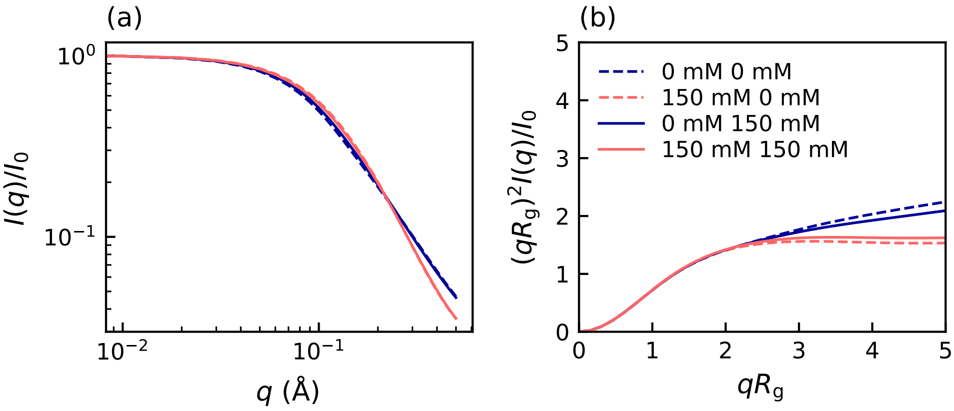

Figure S2: a) Calculated form factor and b) dimensionless Kratky plot of non-phosphorylated (n) and phosphorylated (p) bCPP simulated with 0 or 150 mM NaCl. The data for the phosphorylated peptide are taken from ref. [1].

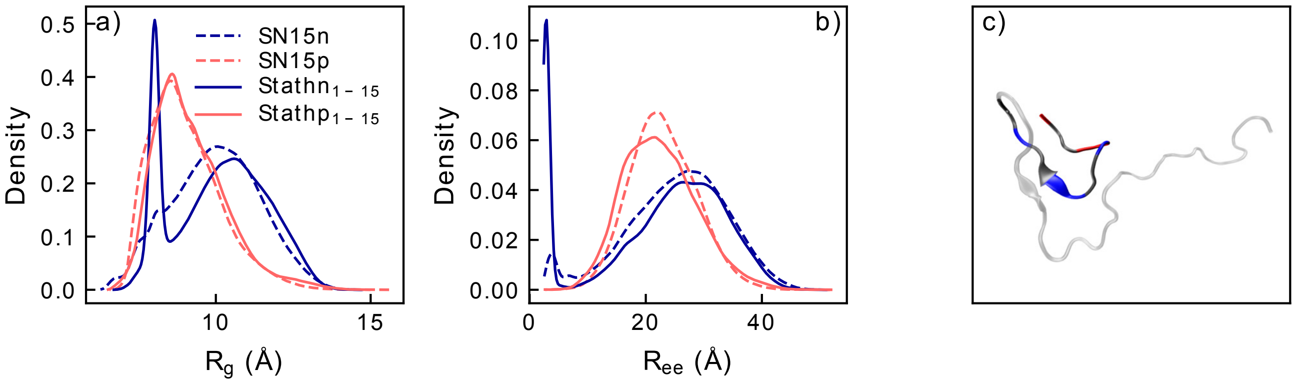

Figure S3: a) Radius of gyration and b) end-to-end distance distributions of non-phosphorylated (n) and phosphorylated (p) Stath$_{1\text{-}15}$ and SN15. c) Snapshot of the type of conformation giving rise to the sharp peak in the Stathn$_{1\text{-}15}$ distributions, where residue 16-43 is traced in light gray. The SN15 data are taken from ref. [2].

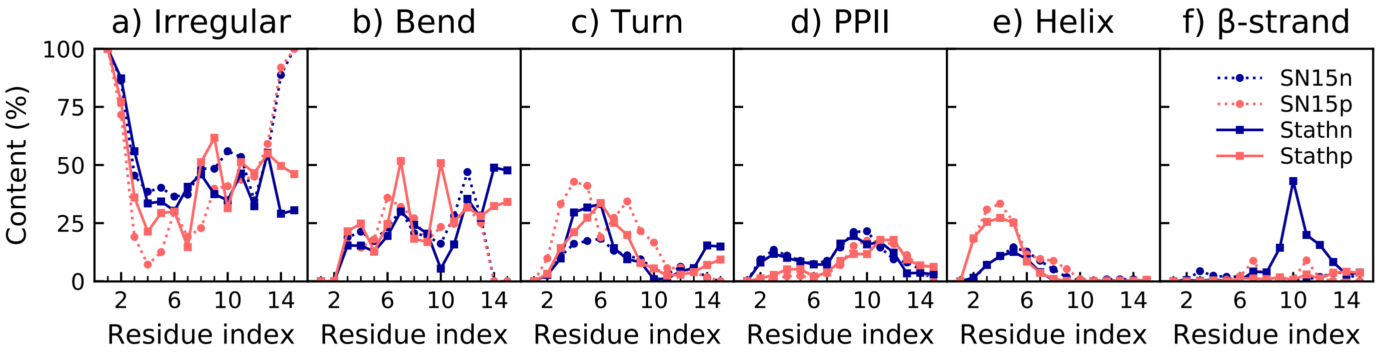

Figure S4: Secondary structure content along the sequence in non-phosphorylated (n) and phosphorylated (p) $Stath_{1\text{-}15}$ and SN15. Helix includes α-helix and 3_{10}-helix. β-strand also includes β-bridge. The SN15 data are taken from ref. [2].

References

[1] Ellen Rieloff and Marie Skepö. Molecular dynamics simulations of phosphorylated intrinsically disordered proteins: A force field comparison. *Int. J. Mol. Sci.*, 22(18), 2021.

[2] Ellen Rieloff and Marie Skepö. Phosphorylation of a disordered peptide—structural effects and force field inconsistencies. *Journal of Chemical Theory and Computation*, 16(3):1924–1935, 2020.
