# Supplementary material for: The Effect of Multisite Phosphorylation on the Conformational Properties of Intrinsically Disordered Proteins
Source: Int J Mol Sci. 2021 Oct 14;22(20):11058. doi: 10.3390/ijms222011058 (PMC8541499; doi:10.3390/ijms222011058)
Supplement: Supplementary file 1 [file ijms-22-11058-s001.zip › SupplementaryMaterial/SupplementaryFileS2.pdf]

Supplementary File S2 for Conformational effects of multisite phosphorylation of disordered peptides

E. Rieloff[1] and M. Skepö[1,2*]

[1] Division of Theoretical Chemistry, Lund University, Lund, Sweden
[2] LINXS - Lund Institute of Advanced Neutron and X-ray Science, Lund, Sweden

* marie.skepo@teokem.lu.se

The time evolution, density distribution, autocorrelation function, and error estimate from block averaging of end-to-end distance and radius of gyration, as well as the energy landscapes constructed from principal component analysis have all been used to assess the convergence and sampling quality of the simulations in this work. For SN15n and SN15p we refer to the supporting information of ref. [1] and for Tau2p, bCPPp, and Stathp we refer to the supplementary material to ref. [2]. The remaining peptides are presented in the following order:

- Tau1n: Figure S1–S3
- Tau1p: Figure S4–S6
- Tau2n: Figure S7–S9
- bCPPn: Figure S10–S12
- bCPPn 150 mM: Figure S13–S14
- Stathn: Figure S15–S17

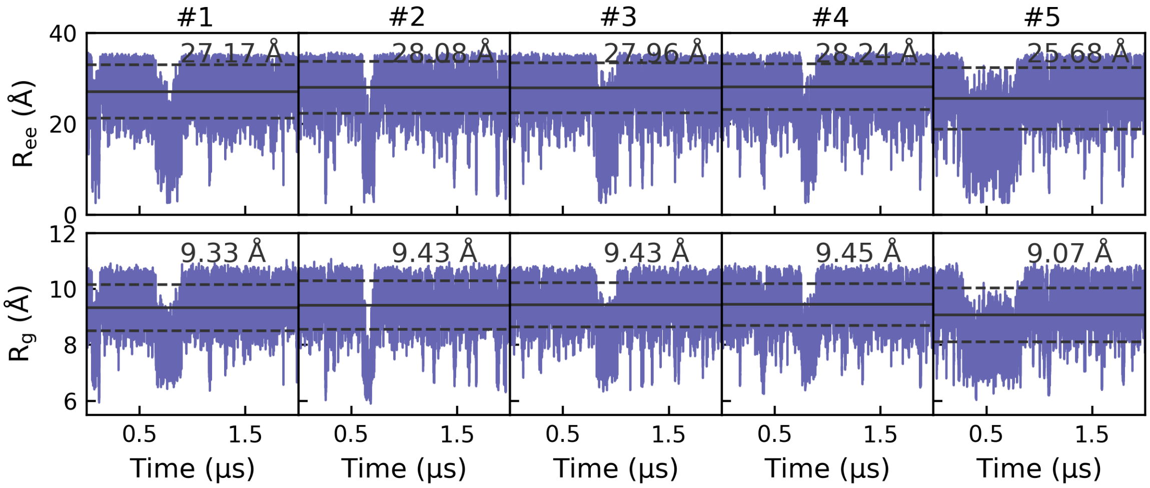

Figure S1: Time evolution of the end-to-end distance (R_{ee}) and the radius of gyration (R_g) for the five replicates in the simulation of Tau1n. The horizontal solid line represents the average in each replicate, with the dashed lines showing the standard deviation.

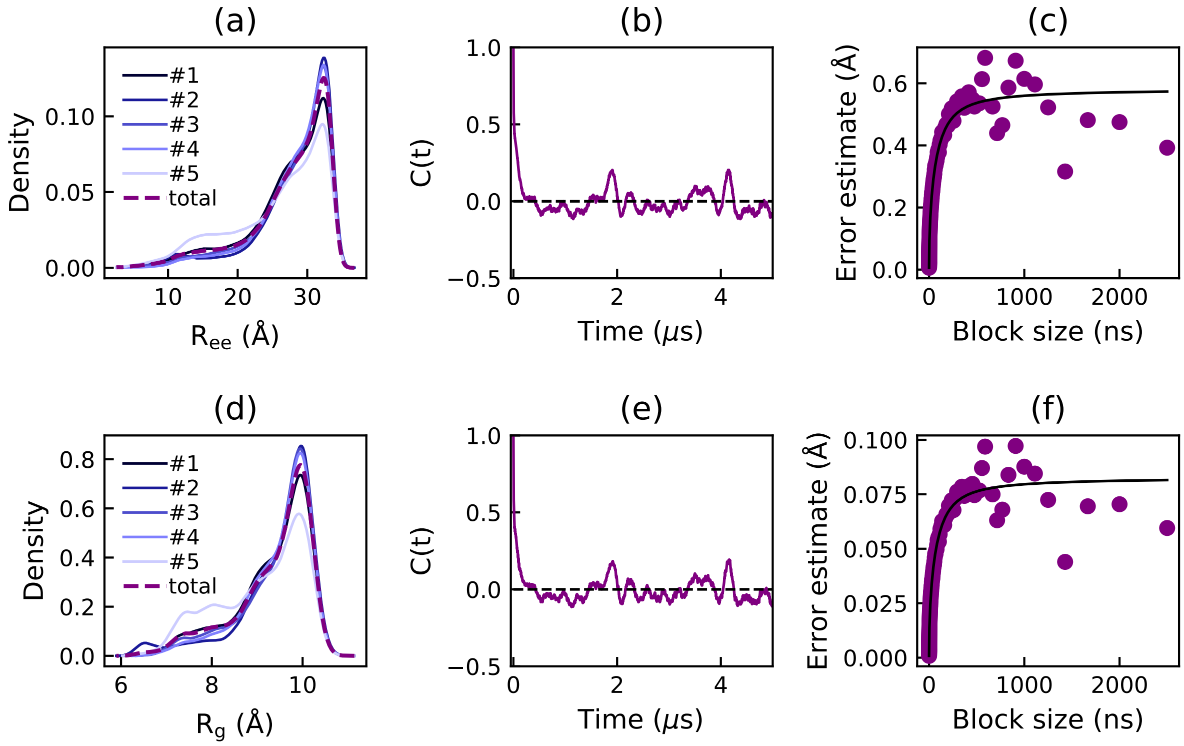

Figure S2: Density estimates of the end-to-end distance (R_{ee}) (a) and the radius of gyration (R_g) (d) for the five replicates and the concatenated simulation of Tau1n, obtained from a Gaussian kernel estimator. Autocorrelation function (C(t)) and error estimate from block averaging of the end-to-end distance (b,c) and the radius of gyration (e,f) for the concatenated simulation.

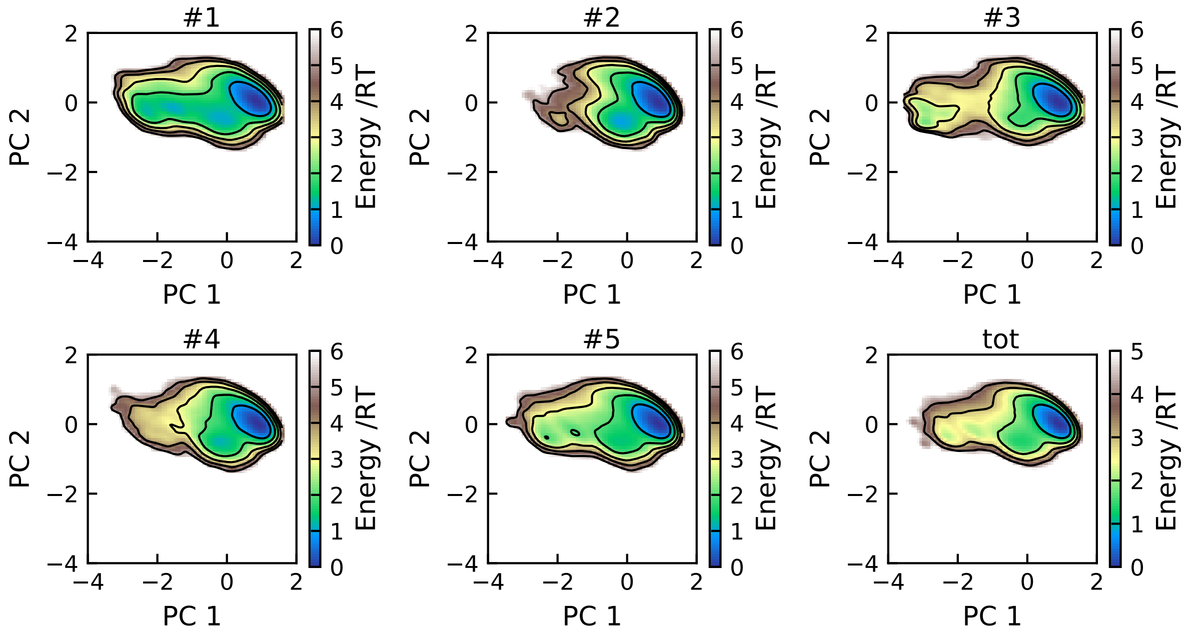

Figure S3: Energy landscapes for the five replicates and the concatenated trajectory of Tau1n, using the first two principal components. All plots have been constructed using the same basis set and are therefore directly comparable. Contour lines are drawn for integer energy levels in the interval $1 \leq RT \leq 5$.

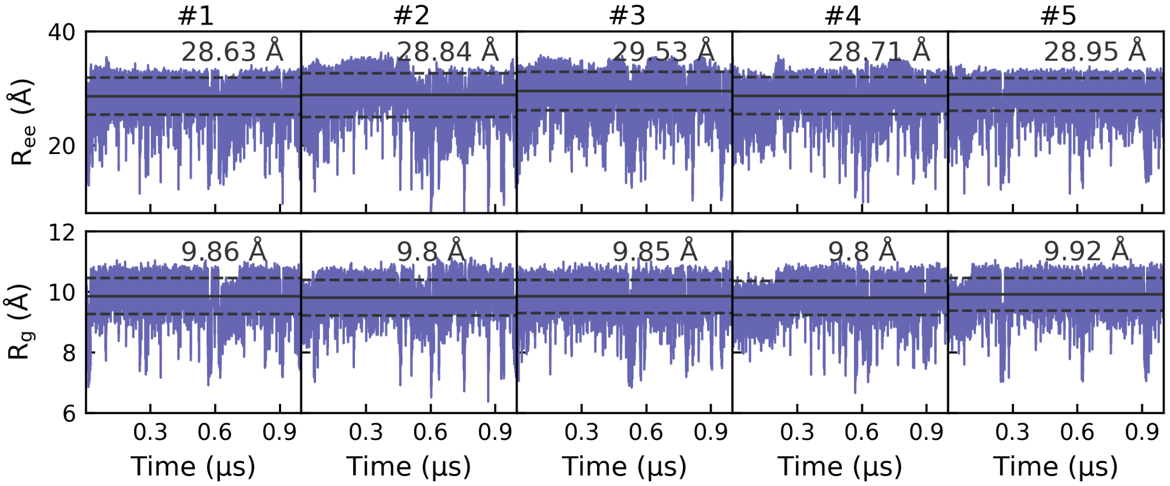

Figure S4: Time evolution of the end-to-end distance (R_{ee}) and the radius of gyration (R_g) for the five replicates in the simulation of Tau1p. The horizontal solid line represents the average in each replicate, with the dashed lines showing the standard deviation.

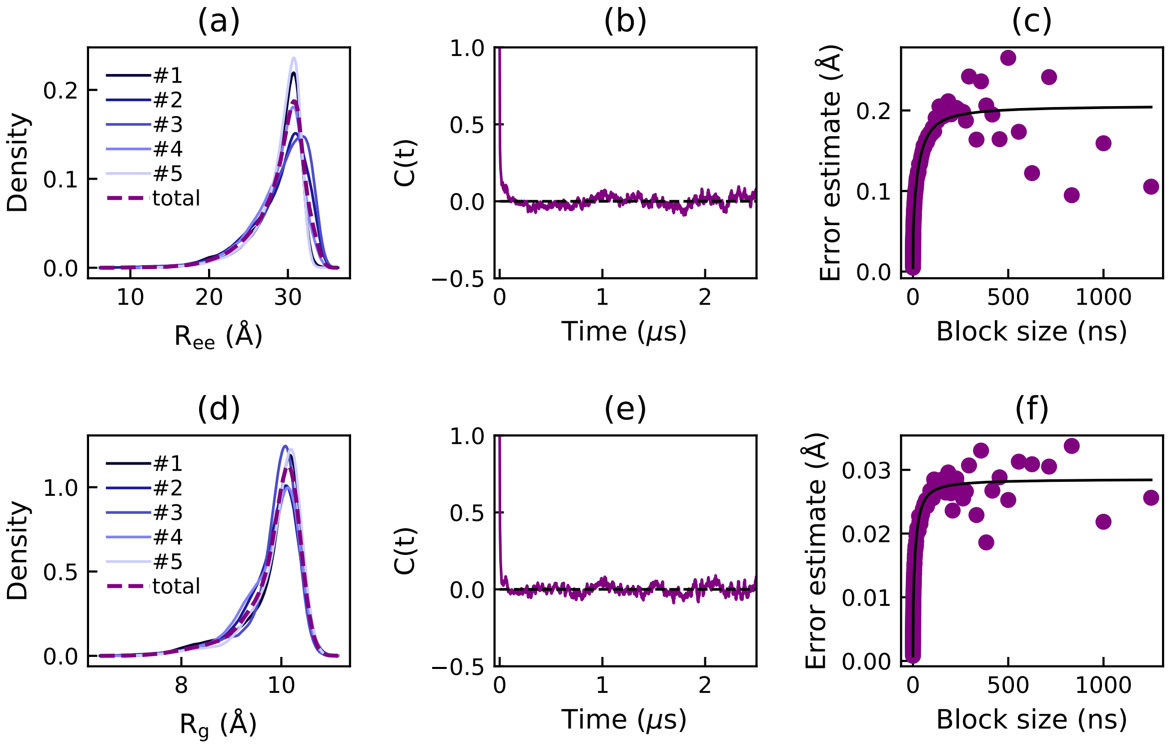

Figure S5: Density estimates of the end-to-end distance (R_{ee}) (a) and the radius of gyration (R_g) (d) for the five replicates and the concatenated simulation of Tau1p, obtained from a Gaussian kernel estimator. Autocorrelation function (C(t)) and error estimate from block averaging of the end-to-end distance (b,c) and the radius of gyration (e,f) for the concatenated simulation.

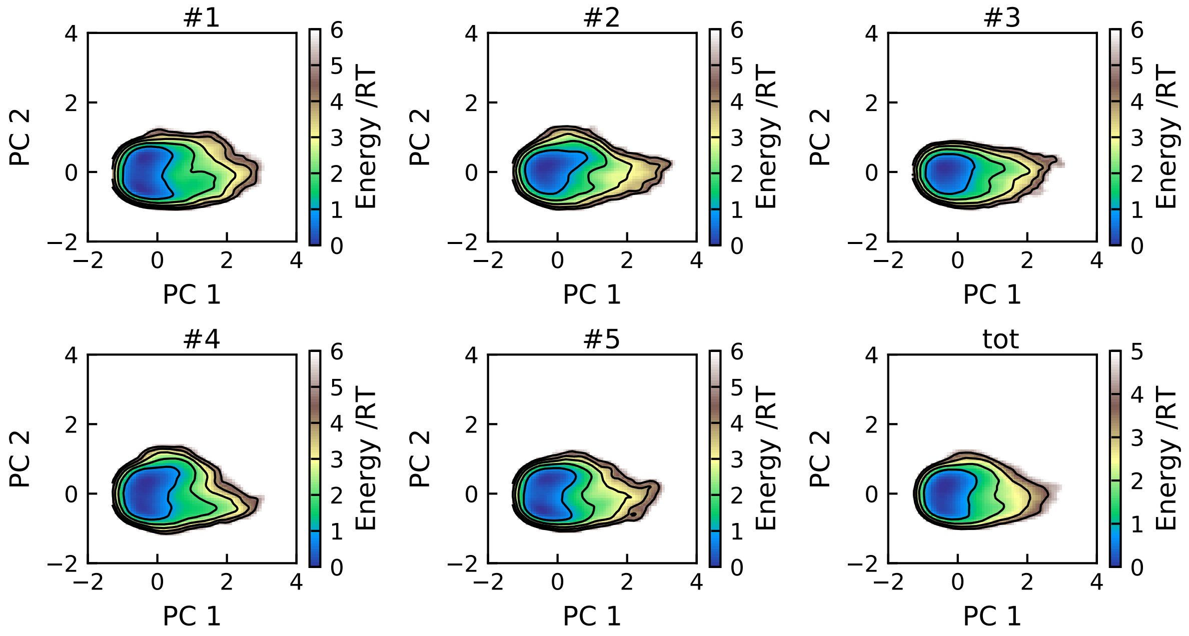

Figure S6: Energy landscapes for the five replicates and the concatenated trajectory of Tau1p, using the first two principal components. All plots have been constructed using the same basis set and are therefore directly comparable. Contour lines are drawn for integer energy levels in the interval $1 \leq RT \leq 5$.

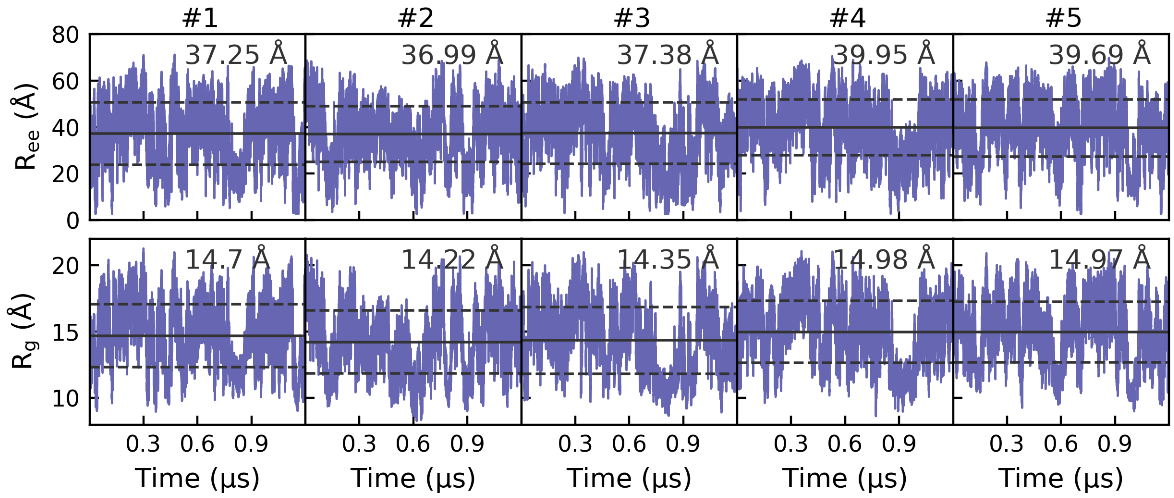

Figure S7: Time evolution of the end-to-end distance (R_{ee}) and the radius of gyration (R_g) for the five replicates in the simulation of Tau2n. The horizontal solid line represents the average in each replicate, with the dashed lines showing the standard deviation.

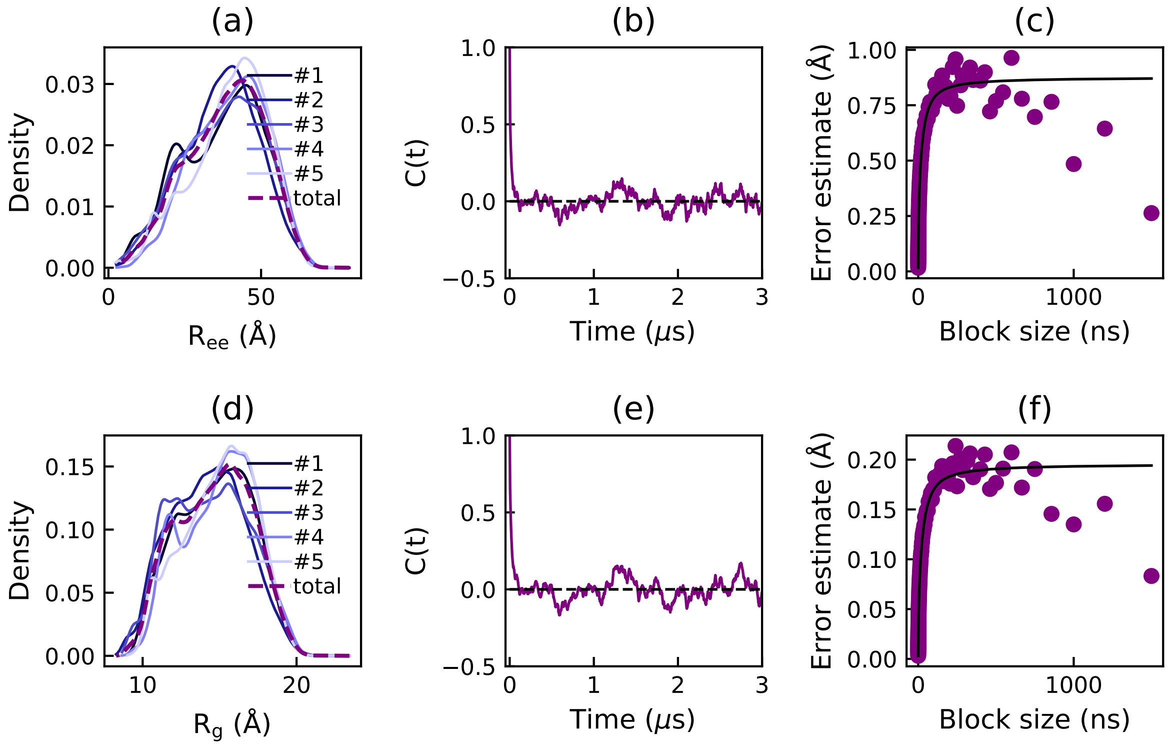

Figure S8: Density estimates of the end-to-end distance (R_{ee}) (a) and the radius of gyration (R_g) (d) for the five replicates and the concatenated simulation of Tau2n, obtained from a Gaussian kernel estimator. Autocorrelation function (C(t)) and error estimate from block averaging of the end-to-end distance (b,c) and the radius of gyration (e,f) for the concatenated simulation.

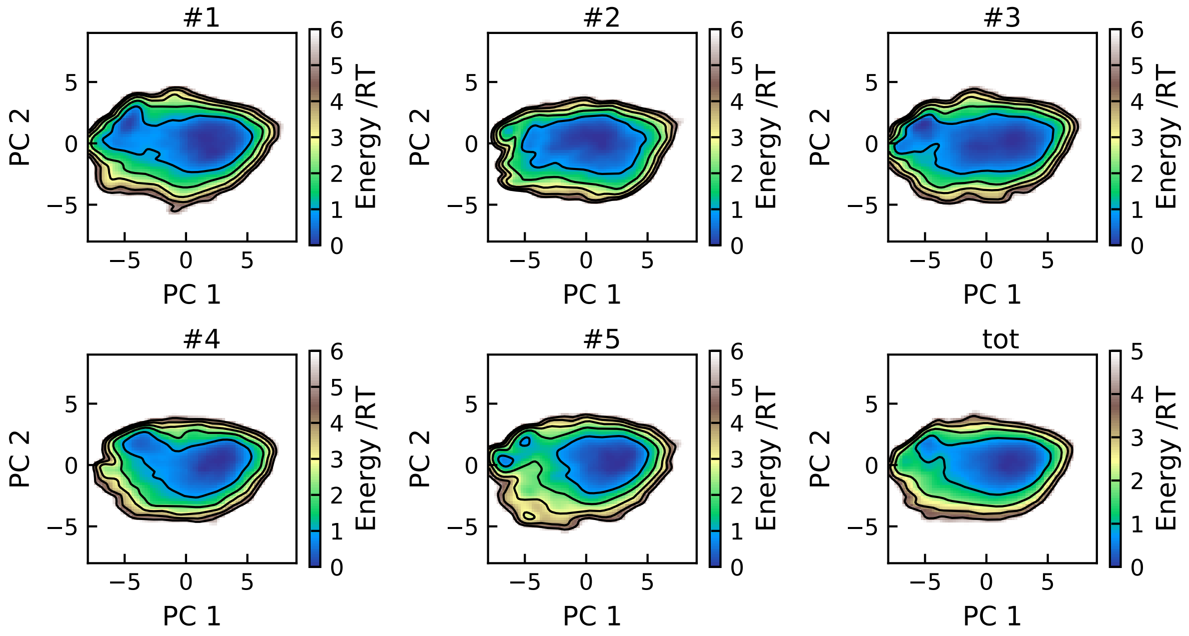

Figure S9: Energy landscapes for the five replicates and the concatenated trajectory of Tau2n, using the first two principal components. All plots have been constructed using the same basis set and are therefore directly comparable. Contour lines are drawn for integer energy levels in the interval $1 \leq RT \leq 5$.

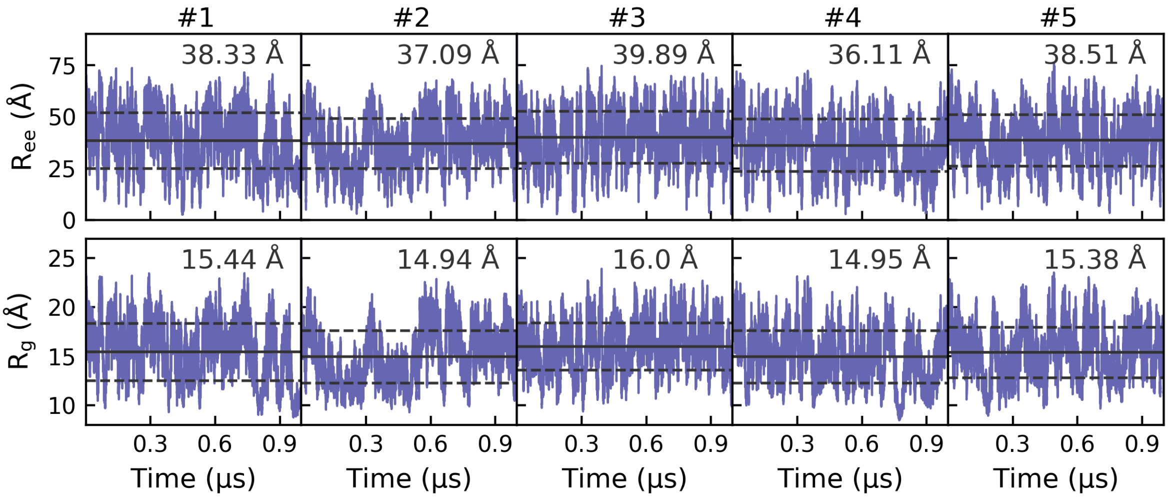

Figure S10: Time evolution of the end-to-end distance (R_{ee}) and the radius of gyration (R_g) for the five replicates in the simulation of bCPPn. The horizontal solid line represents the average in each replicate, with the dashed lines showing the standard deviation.

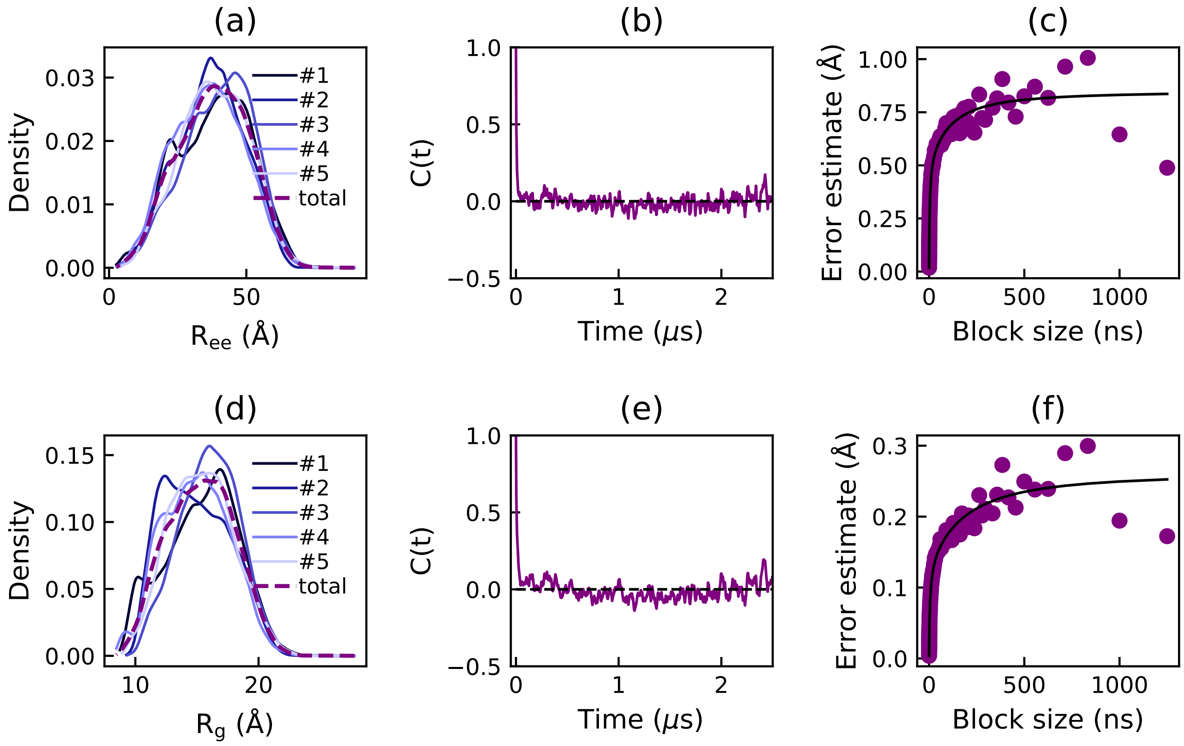

Figure S11: Density estimates of the end-to-end distance (R_{ee}) (a) and the radius of gyration (R_g) (d) for the five replicates and the concatenated simulation of bCPPn, obtained from a Gaussian kernel estimator. Autocorrelation function (C(t)) and error estimate from block averaging of the end-to-end distance (b,c) and the radius of gyration (e,f) for the concatenated simulation.

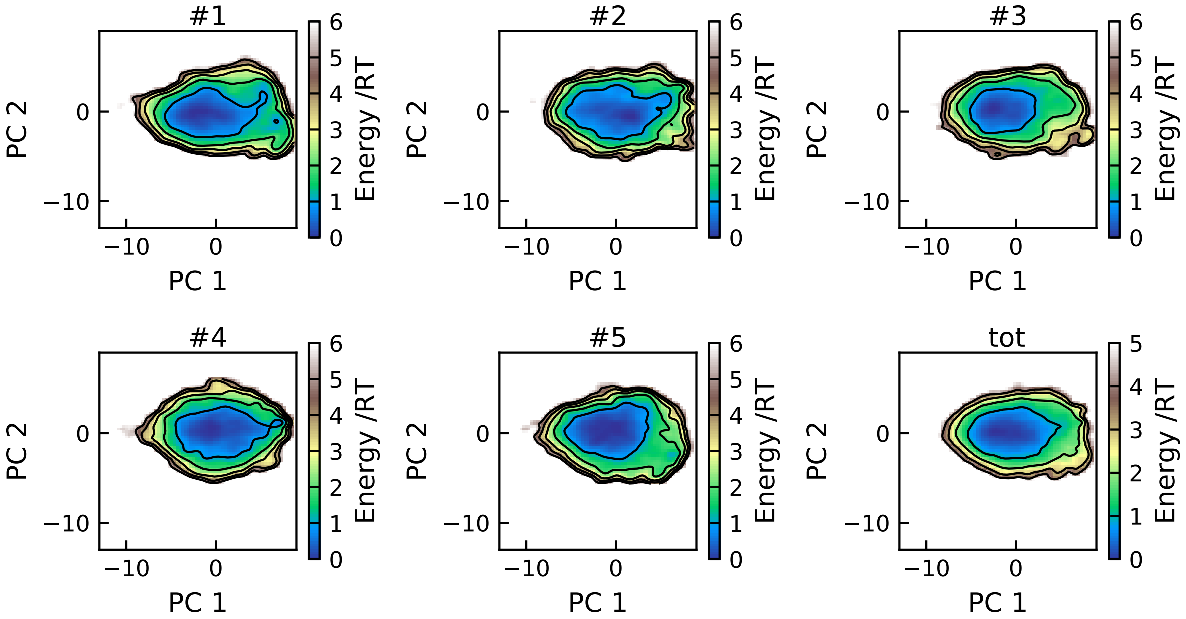

Figure S12: Energy landscapes for the five replicates and the concatenated trajectory of bCPPn, using the first two principal components. All plots have been constructed using the same basis set and are therefore directly comparable. Contour lines are drawn for integer energy levels in the interval $1 \leq RT \leq 5$.

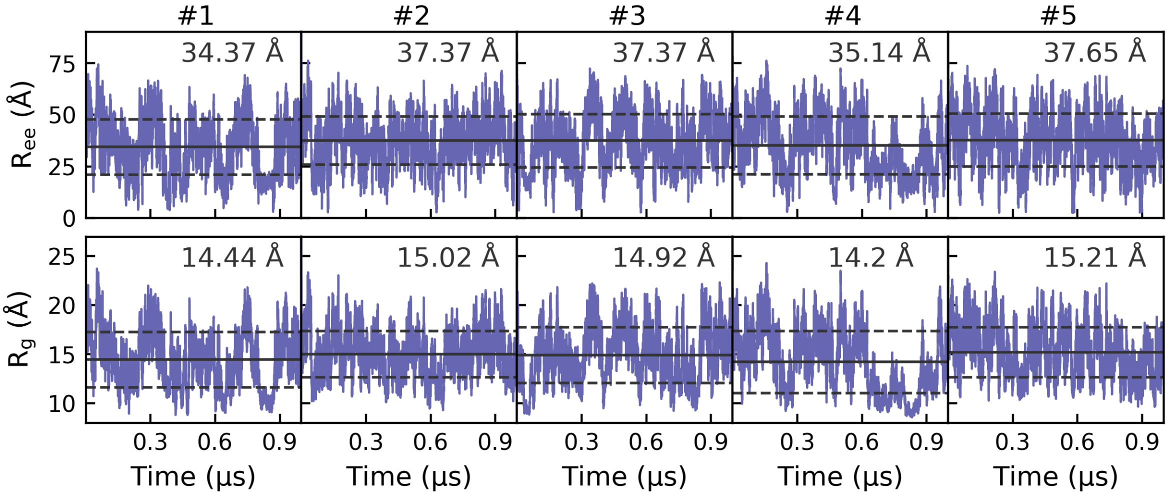

Figure S13: Time evolution of the end-to-end distance (R_{ee}) and the radius of gyration (R_g) for the five replicates in the simulation of bCPPn with 150 mM NaCl. The horizontal solid line represents the average in each replicate, with the dashed lines showing the standard deviation.

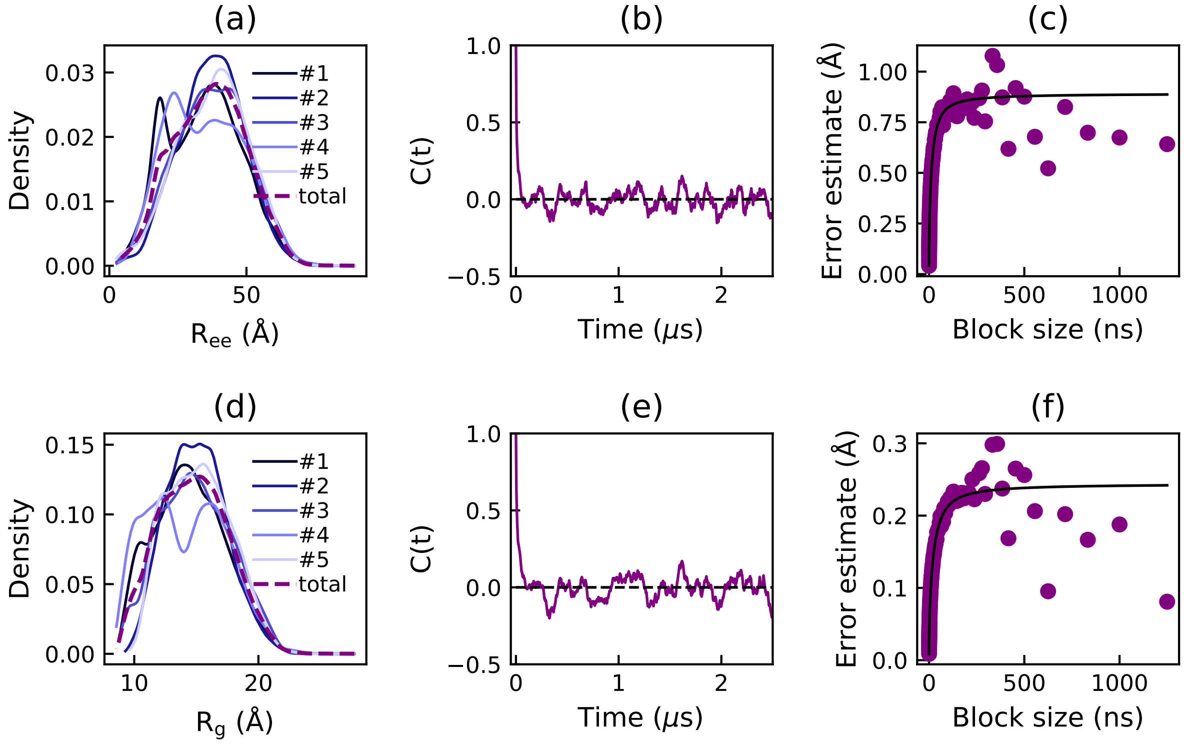

Figure S14: Density estimates of the end-to-end distance (R_{ee}) (a) and the radius of gyration (R_g) (d) for the five replicates and the concatenated simulation of bCPPn with 150 mM NaCl, obtained from a Gaussian kernel estimator. Autocorrelation function (C(t)) and error estimate from block averaging of the end-to-end distance (b,c) and the radius of gyration (e,f) for the concatenated simulation.

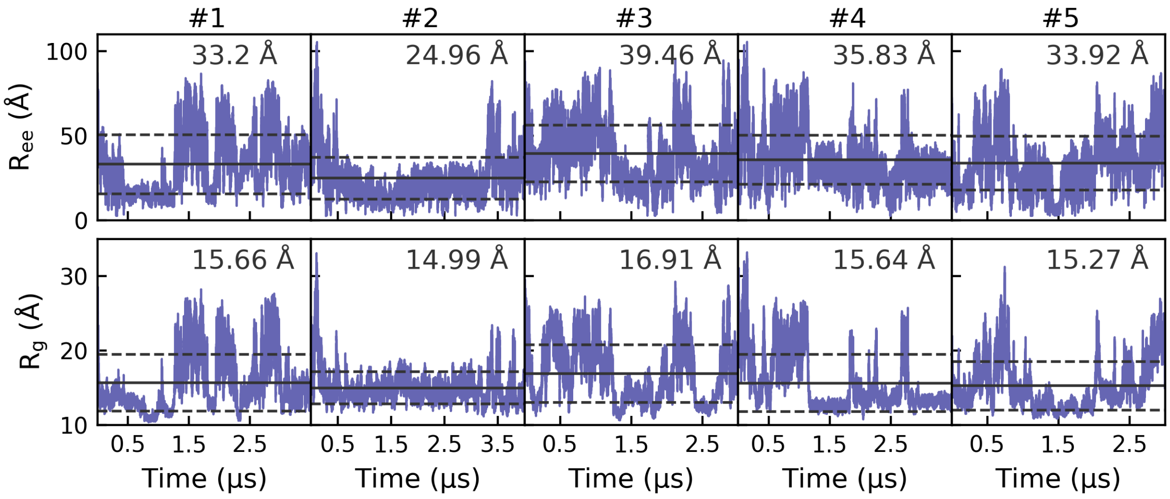

Figure S15: Time evolution of the end-to-end distance (R_{ee}) and the radius of gyration (R_g) for the five replicates in the simulation of Stathn. The horizontal solid line represents the average in each replicate, with the dashed lines showing the standard deviation.

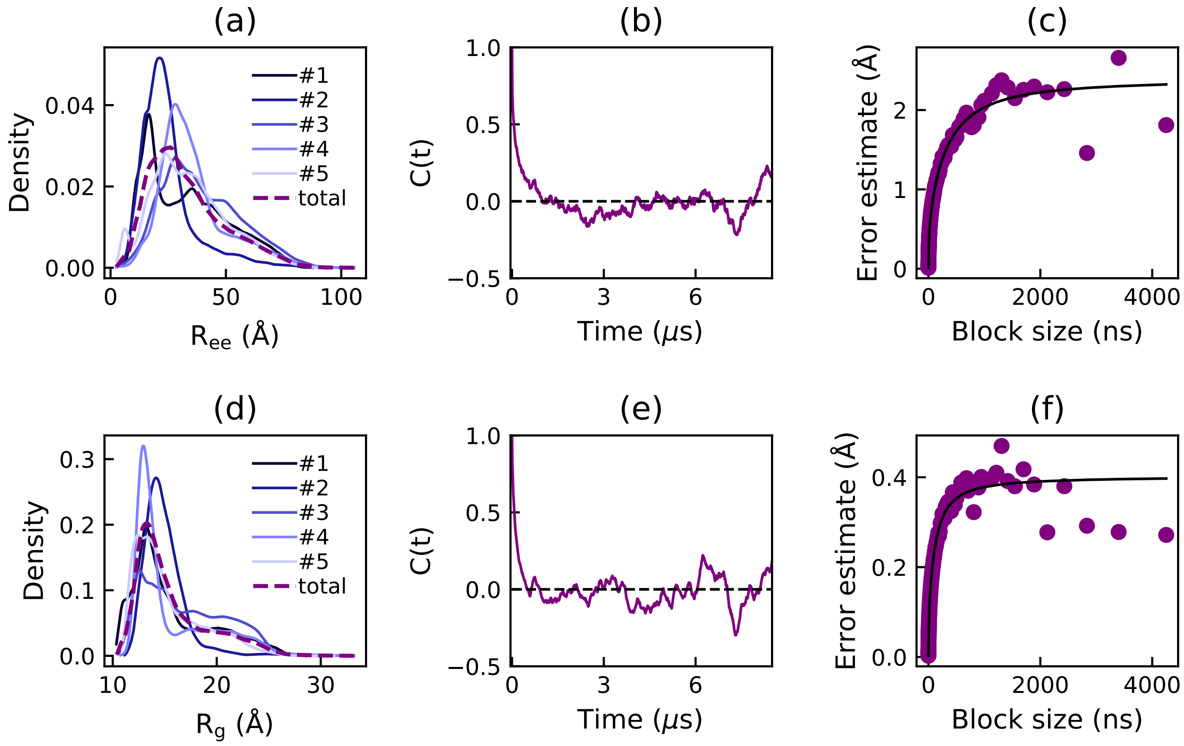

Figure S16: Density estimates of the end-to-end distance (R_{ee}) (a) and the radius of gyration (R_g) (d) for the five replicates and the concatenated simulation of Stathn, obtained from a Gaussian kernel estimator. Autocorrelation function (C(t)) and error estimate from block averaging of the end-to-end distance (b,c) and the radius of gyration (e,f) for the concatenated simulation.

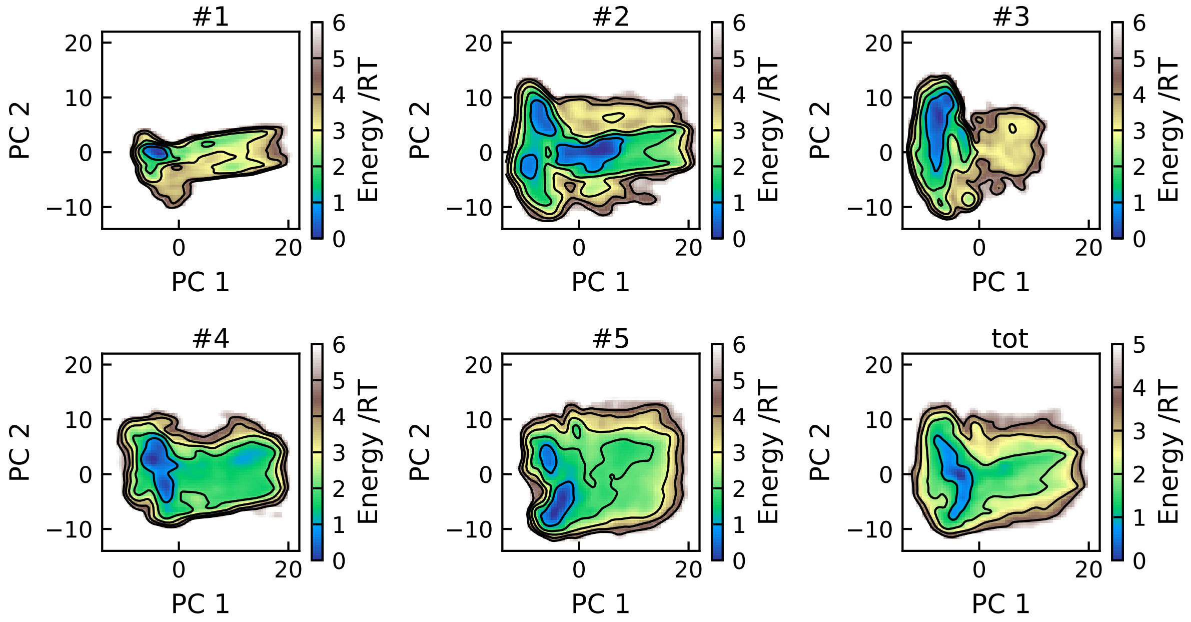

Figure S17: Energy landscapes for the five replicates and the concatenated trajectory of Stathn, using the first two principal components. All plots have been constructed using the same basis set and are therefore directly comparable. Contour lines are drawn for integer energy levels in the interval $1 \leq RT \leq 5$.

References

[1] Ellen Rieloff and Marie Skepö. Phosphorylation of a disordered peptide—structural effects and force field inconsistencies. *J. Chem. Theory Comput.*, 16(3):1924–1935, 2020.

[2] Ellen Rieloff and Marie Skepö. Molecular dynamics simulations of phosphorylated intrinsically disordered proteins: A force field comparison. *Int. J. Mol. Sci.*, 22(18), 2021.
